# Association of work-time control with burnout and turnover intention: a cross-sectional analysis of a general working population in Korea

**DOI:** 10.4178/epih.e2026011

**Published:** 2026-02-21

**Authors:** Hye-Eun Lee, Seong-Sik Cho, Mo-Yeol Kang

**Affiliations:** 1 Department of Social and Preventive Medicine, Hallym University College of Medicine, Chuncheon, Korea; 2 Institute of Social Medicine, Hallym University College of Medicine, Chuncheon, Korea; 3 Department of Occupational and Environmental Medicine, Dong-A University College of Medicine, Busan, Korea; 4 Department of Occupational and Environmental Medicine, College of Medicine, The Catholic University of Korea, Seoul, Korea

**Keywords:** Occupational stress, Burnout, professional, Personnel turnover, Occupational health

## Abstract

**OBJECTIVES:**

For employees, work-time control (WTC) may protect against burnout and turnover. However, evidence from Korean workplaces is limited. This study aimed to examine whether WTC is associated with burnout and turnover intention and to test whether burnout mediates this relationship.

**METHODS:**

We analyzed data from 4,745 wage workers in the 2024 wave of the Korean Work, Sleep, and Health Study. WTC was assessed across 6 domains, burnout was measured using the Korean Burnout Syndrome Scale, and turnover intention was assessed using a validated 4-item scale. Logistic regression was used to estimate the associations of WTC quartile with burnout and turnover intention, and mediation analysis was used to decompose the association between WTC and turnover intention through burnout.

**RESULTS:**

Among 4,745 workers, the prevalence of burnout was 3.9% and turnover intention was 34.5%; both increased stepwise across lower WTC quartiles. In adjusted models, workers in the lowest WTC quartile had higher odds of burnout (odds ratio [OR], 3.95; 95% confidence interval [CI], 2.41 to 6.47) and turnover intention (OR, 2.24; 95% CI, 1.85 to 2.71) than those in the highest quartile. Mediation analysis showed that burnout explained 36.6% (95% CI, 22.3 to 51.0) of the association between WTC and turnover intention.

**CONCLUSIONS:**

Lower WTC was linked to higher burnout and turnover intention, with burnout explaining more than one-third of this relationship.

## GRAPHICAL ABSTRACT

[Fig f1-epih-48-e2026011]

## Key Message

Lower work-time control (WTC) was associated with higher burnout and greater turnover intention among Korean workers. Workers in the lowest WTC quartile had nearly four times higher odds of burnout and more than twice the odds of turnover intention compared with those in the highest quartile. Burnout mediated about 36.6% of the association between WTC and turnover intention, suggesting that improving schedule control may reduce turnover partly by preventing burnout.

## INTRODUCTION

Burnout has become a critical public health and organizational concern, with substantial implications for individual well-being and economic productivity [1]. Characterized by emotional exhaustion, cynicism, and reduced professional efficacy, burnout has been linked to a range of adverse health outcomes, including cardiovascular disease, depression, and increased overall mortality risk [2-4]. From an economic perspective, burnout reduces job performance, increases absenteeism, and increases turnover rates, imposing substantial costs on both employers and the healthcare system [5-7].

Burnout not only undermines individual health but also shapes employees’ organizational attitudes, most notably by increasing turnover intention [8]. Turnover intention refers to an employee’s conscious and deliberate willingness to leave the organization and is a widely recognized predictor of actual turnover behavior [9]. High turnover intention threatens organizational stability by increasing the risk of workforce loss, operational disruption, and recruitment costs [10]. These concerns underscore the importance of identifying workplace factors that can reduce burnout and turnover intention for employers and policymakers.

Work-time control (WTC)—the extent to which employees can influence the timing, duration, and distribution of their working hours—is considered a component of job autonomy within the job demands–control (JD-C) model [11]. Greater WTC helps employees align work demands with personal needs, increases opportunities for recovery (e.g., sufficient sleep and psychological detachment), and reduces work–family conflict, thereby lowering the risk of emotional exhaustion [12-14]. In addition, the job demands–resources (JD-R) model provides a robust framework for conceptualizing WTC as a critical “job resource.” In the JD-R model, job resources function as buffers that reduce the physiological and psychological costs associated with high job demands [15]. Integrating these perspectives provides a strong rationale for the hypothesis that burnout is a key psychological mechanism linking limited work-time autonomy to increased intention to quit among Korean workers.

Although WTC has been linked to multiple outcomes—including sleep quality, work–life balance, job satisfaction, and general mental health [12,16-18]—its direct association with burnout has received comparatively little attention. Evidence on WTC and turnover intention exists [19-21], but it is largely confined to Western intervention contexts and healthcare settings. Korean workplaces are characterized by long working hours and rigid hierarchical control, which constrains employees’ ability to shape when and how long they work [22]. Although legal limits on weekly hours (52 hr/wk) in Korea have provided benefits in certain cases, excessive workloads persist because of uneven coverage and ongoing presenteeism.

Thus, Korea is an appropriate context for examining whether WTC is associated with burnout and turnover intention. Moreover, integrated tests of the pathway from WTC to turnover intention via reduced burnout are scarce. Accordingly, this study examined the associations among WTC, burnout, and turnover intention and tested whether burnout mediated the WTC–turnover intention relationship in Korea using large-scale data spanning diverse occupations. We hypothesized that higher WTC would be associated with lower burnout and turnover intention and that burnout would partially mediate the association between WTC and turnover intention.

## MATERIALS AND METHODS

### Study population

We analyzed data from the Korean Work, Sleep, and Health Study (KWSHS), a national longitudinal survey initiated in 2022 to examine links among working conditions, sleep, and health in Korean workers. The panel completed 5 waves approximately every 6 months from July 2022 to September 2024. Eligible participants were economically active adults aged 19–70 years from diverse industries [23]. The present analysis used data from the final survey conducted in September 2024, which included the WTC assessment. Of the 5,783 respondents, we excluded 446 self-employed or unpaid family workers and 592 cases with missing key variables, yielding a final analytic sample of 4,745 participants.

### Work-time control

In accordance with Ala-Mursula et al. [16], we operationalized WTC as perceived influence over 6 domains of working hours: (1) start/end times of the workday, (2) opportunities to take breaks, (3) opportunities to handle private matters during work, (4) scheduling of shifts, (5) scheduling of vacations/paid days off, and (6) taking unpaid leave. Items were rated on a 5-point Likert scale (1=very little to 5=very much). Scores were summed (range, 6–30) to represent overall WTC, with higher scores indicating greater control. Participants were categorized into 4 groups based on quartiles of the total WTC score (Q1–Q4, highest to lowest).

### Burnout

Burnout was measured using the Korean Burnout Syndrome Scale (KBOSS), which was developed to align with the World Health Organization’s International Classification of Diseases, 11th revision (ICD-11) conceptualization of burnout as a work-related syndrome comprising 3 dimensions: exhaustion, cynicism/mental distance, and reduced professional efficacy [24]. The KBOSS contains 12 items (4 per dimension) rated on a 7-point Likert scale (1=strongly disagree to 7=strongly agree), with higher scores indicating greater symptom burden. For each respondent, we computed dimension scores by summing the 4 items within each domain (possible range, 4–28 per dimension). Following the instrument’s validation study, we also created a binary burnout classification: respondents meeting all 3 dimension-specific cutoffs (exhaustion ≥21, cynicism ≥18, inefficacy ≥15) were classified as having burnout. The KBOSS demonstrated good internal consistency in the validation study (overall α=0.813) [24].

### Turnover intention

Turnover intention was assessed using the Korean-translated version of Lawler’s 4-item instrument, which has been widely used in Korea [25,26]. Each item was rated on a 5-point Likert scale (1=“not at all” to 5=“strongly agree”), and item scores were summed to yield a total score ranging from 4 to 20, with higher values indicating stronger turnover intention. We created a binary indicator in which total scores ≥16 (i.e., a mean item score ≥4) were coded as turnover intention present, consistent with prior research defining high turnover intention as endorsement of at least “agree” on most items of the scale [27].

### Covariates

Our statistical models were adjusted for gender, age, educational attainment, monthly salary, occupation, weekly working hours, and shift work; all covariates were obtained from the survey. Education was dichotomized as ≤high school versus ≥college. Monthly salary was categorized as <Korean won [KRW] 2.00 million, 2.00–2.99 million, 3.00–3.99 million, and ≥4.00 million. Occupation was grouped as white collar (managers, professionals, clerical), pink collar (service, sales), or blue collar (craft, manual). Weekly working hours were classified as <40, 40–52, and >52 hr/wk in line with the Korean Labor Standards Act (a 40-hour standard workweek plus up to 12 hours of overtime) [28]. Shift work was defined based on a survey item asking whether participants primarily worked outside daytime hours (6:00 A.M. to 6:00 P.M.).

### Statistical analysis

We first described overall participant characteristics and the prevalence of burnout and turnover intention, then compared socio-demographic and job-related characteristics across WTC quartile groups using chi-square tests. Associations between WTC quartiles and each binary outcome (burnout and turnover intention) were estimated using logistic regression, reporting odds ratios (ORs) and 95% confidence intervals (CIs), with Q1 (the highest WTC quartile) as the reference group. We present unadjusted results and 2 adjusted models: model 1 was adjusted for age and gender, while model 2 was additionally adjusted for education, monthly salary, occupational class, weekly working hours, and shift work.

Stratified logistic regression analyses by gender, age group, and weekly working hours were performed to explore potential heterogeneity in the associations between WTC and burnout or turnover intention. Sensitivity analyses were conducted using alternative burnout definitions, including a less stringent binary definition (meeting at least 2 of the 3 burnout dimensions) and a continuous burnout score based on the total KBOSS. Logistic and linear regression models with the same covariate structure as the main analyses were applied. To examine potential heterogeneity across different aspects of WTC, we also conducted sensitivity analyses for each of the 6 individual WTC domains. Each domain was analyzed separately as a continuous variable, with ORs estimated per 1-point decrease on the original 5-point Likert scale.

For the mediation analysis, we dichotomized WTC to enhance interpretability and statistical power, defining higher control as Q1–Q2 and lower control as Q3–Q4. Using a counterfactual framework, we decomposed the total effect of WTC on turnover intention into natural direct and indirect effects operating through burnout (a binary mediator). We reported total, direct, and indirect ORs along with the percentage mediated and corresponding 95% CIs. We additionally conducted mediation analyses treating WTC as a continuous variable using the total score, with ORs estimated per 1-point decrease in WTC and adjustment for the same covariates as in the main analyses.

Baseline characteristics of included and excluded participants were compared to assess potential selection bias due to missing data. All analyses used two-sided tests, with statistical significance set at p-value <0.05. Statistical analyses were conducted using SAS OnDemand for Academics (SAS Institute Inc., Cary, NC, USA). Counterfactual mediation analyses were performed using the PROC CAUSALMED procedure in SAS.

### Ethics statement

This study complied with the principles of the Declaration of Helsinki. Informed consent was obtained from all the participants, and anonymity and confidentiality were ensured. The Institutional Review Board of Dong-A University approved the study protocol (IRB No. 2-1040709-AB-N-01-202202-HR-017-06).

## RESULTS

Table 1 presents the baseline characteristics of participants by WTC quartile. Lower levels of WTC were more frequently observed among women, older workers, individuals with lower educational attainment and income, those in blue-collar occupations, and those engaged in shift work (all p<0.05). Among the 4,745 workers, the prevalence of burnout was 3.9% (n=187), and turnover intention was 34.5% (n=1,638). Both outcomes increased stepwise with lower WTC, from 2.2% and 27.4% in Q1 (the highest WTC) to 7.3% and 44.9% in Q4 (the lowest WTC) (p<0.001 for both). Prevalence of burnout was higher among women and in the youngest age groups. Turnover intention displayed a similar age gradient and was also more prevalent among women. Weekly working hours were modestly associated with turnover intention (31.2% for <40 hr/wk, 34.9% for 40–52 hr/wk, and 39.1% for >52 hr/wk; p=0.05), whereas education and shift work displayed no clear associations.

As shown in Table 2, in the fully adjusted model (model 2), lower WTC was associated with progressively higher odds of burnout: Q2 (adjusted odds ratio [aOR], 1.37; 95% CI, 0.80 to 2.34); Q3 (aOR, 1.80; 95% CI, 1.07 to 3.03); and Q4 (aOR, 3.95; 95% CI, 2.41 to 6.47) compared with Q1 (reference). For turnover intention, adjusted odds also increased with lower WTC: Q2 (aOR, 1.15; 95% CI, 0.95 to 1.38); Q3 (aOR, 1.53; 95% CI, 1.27 to 1.84); and Q4 (aOR, 2.24; 95% CI, 1.85 to 2.71) versus Q1 (reference).

Stratified analyses showed broadly consistent associations between lower WTC and both burnout and turnover intention across subgroups defined by gender, age, and weekly working hours (Supplementary Materials 1 and 2).

Sensitivity analyses using alternative burnout definitions displayed patterns consistent with the main findings, with higher burnout among workers with lower WTC, despite some heterogeneity across intermediate categories (Supplementary Materials 3 and 4).

All 6 WTC domains were significantly associated with burnout and turnover intention, with relatively stronger associations for domains related to shift scheduling and handling of private matters during work (Supplementary Material 5).

Mediation analysis (Table 3) indicated that burnout accounted for a substantial share of the association between WTC and turnover intention: the total effect odds ratio (OR) was 1.96 (95% CI, 1.64 to 2.28), the natural direct effect OR was 1.61 (95% CI, 1.40 to 1.82), and the natural indirect effect OR was 1.22 (95% CI, 1.09 to 1.34). Overall, 36.6% (95% CI, 22.3 to 51.0) of the association was mediated by burnout after covariate adjustment. Mediation analyses treating WTC as a continuous variable yielded results consistent with the main findings, with burnout mediating approximately one-third of the association with turnover intention (Supplementary Material 6).

Baseline characteristics of participants included in the analytic sample were compared with those excluded due to missing data (Supplementary Material 7). Excluded participants were more likely to have higher education levels, higher monthly salaries, white-collar occupations, and non-shift work, whereas the distributions of age, gender, and weekly working hours were generally similar between the 2 groups.

## DISCUSSION

In this large cross-sectional analysis of Korean workers, lower WTC was associated with higher burnout and greater turnover intention, with a clear dose–response pattern across WTC levels. These relationships persisted after adjustment for socio-demographic and job-related factors. The findings were further supported by sensitivity and stratified analyses, which showed generally consistent patterns across alternative burnout definitions and key subgroups. Domain-specific analyses suggested that domains related to shift scheduling and handling of private matters during work may be especially relevant, although all domains showed significant associations.

Our findings are consistent with prior evidence that greater schedule control is associated with lower turnover intention. In a natural experiment among corporate employees at a large United States company, expanding employees’ control over work schedules was associated with lower turnover intention [19]. Similar patterns have been reported among United States nursing home staff, in German survey analyses of healthcare workers, and among registered nurses in Swiss psychiatric hospitals, where higher WTC was associated with lower turnover intention [20,21,29].

Importantly, our study extends the literature by demonstrating that burnout partially mediated the association between WTC and turnover intention. This suggests that WTC may reduce turnover not only directly, through increased schedule predictability and autonomy, but also indirectly by alleviating exhaustion, a proximal determinant of quitting intentions. Consistent with our findings, a 3-wave German panel study reported that WTC was indirectly associated with lower exhaustion through reduced internal work-to-home interference [30]. Similarly, in a sample of 2,248 Swedish knowledge workers, control over time was significantly associated with lower exhaustion and reduced work–life interference, particularly among women [14]. In contrast, a study of Swiss psychiatric nurses found that perceived WTC predicted turnover intention but was not significantly associated with emotional exhaustion [29]. Likewise, a small study of Chinese knowledge employees did not identify a significant association between WTC and burnout, instead finding that WTC exerted an impact on innovation through job engagement [31]. Although most prior studies have focused on exhaustion—a core dimension of burnout—our study assessed burnout as a multidimensional construct.

The present findings can be interpreted within both the JD-C and JD-R frameworks. In the classical JD-C model, WTC represents a form of decision latitude that reflects employees’ ability to influence when they work and how much overtime they perform, thereby reducing strain by mitigating the impact of excessive demands [32]. In turn, this may lower the risk of burnout and subsequent turnover intention. In the more recent JD-R model, WTC is conceptualized as a job resource that buffers high demands and facilitates recovery and motivation [15]. Thus, WTC may protect employees from exhaustion and reduce their intention to leave the organization. From the perspective of effort–recovery theory, WTC supports both internal recovery (short breaks and adjustments during work) and external recovery, which occurs outside working hours through sufficient rest, leisure, and psychological detachment from work [33]. By facilitating these recovery processes, WTC can reduce the risk of burnout and thus lower employees’ intention to leave. From a social exchange perspective, organizations that provide employees with greater schedule control may elicit stronger perceptions of organizational support and reciprocity, thereby reducing employees’ intention to leave [34]. Finally, work–life interface theories suggest that WTC facilitates better alignment between work and non-work domains, reducing interference and protecting against both burnout and turnover intention [35].

Beyond these theoretical implications, our findings have important implications for organizational practices and labor policies. At the organizational level, expanding workers’ ability to choose when to start and end their shifts, negotiating or swapping schedules, and reducing involuntary overtime—along with supportive scheduling practices from supervisors—are practical interventions that may improve retention and employee well-being. Supervisors play a central role in shaping cultural norms around scheduling, legitimizing employees’ use of temporal autonomy, and ensuring that flexible policies are implemented effectively. A study of Chinese nurses showed that supervisor support significantly improves job and life satisfaction, in part by facilitating employee-driven flexible working hours [36]. For managers, fostering a workplace culture that respects temporal autonomy may be particularly important in Korea, where long working hours and hierarchical control are prevalent.

Specifically, the higher prevalence of burnout among pink-collar workers and the elevated turnover intention among workers in their 20s and 30s provide insight into how WTC may operate in contemporary Korean workplaces. Service and sales occupations are characterized by high emotional labor demands and limited discretion over work schedules; in Korea, sustained emotional regulation under rigid scheduling conditions may contribute to greater exhaustion and burnout. At the same time, younger workers increasingly emphasize work–life balance, psychological well-being, and autonomy at work, and rigid working-time arrangements may exacerbate misalignment between organizational practices and these expectations, thereby increasing turnover intention. In this context, the lack of WTC may contribute to “quiet quitting” as an adaptive response to structural workplace constraints [37]. From this perspective, WTC may function as a critical job resource that buffers emotional strain and supports better alignment between work demands and evolving workforce expectations, particularly in emotionally demanding occupations and among younger workers.

International experience suggests that stronger institutional support for WTC is feasible. For example, under the Employment Rights Act 1996 (Part 8A), employees in the United Kingdom have a statutory right to request flexible working arrangements—including changes to start and end times, total hours, or work location—from the first day of employment [38]. Scandinavian countries provide broader employee-centered rights, with extensive flexible-work options and a wide range of family-friendly arrangements that extend beyond parental leave, thereby enhancing workers’ capacity to reconcile occupational and non-work demands [39].

In contrast, Korea’s Labor Standards Act provides flexible working-hour arrangements, such as flexible and discretionary working schemes. These provisions allow employers to average working hours over reference periods or set total hours within a settlement period, thus granting operational flexibility. However, the law does not explicitly grant employees the right to determine or request their schedules, and in practice, such schemes often operate primarily according to employers’ needs, increasing irregularity and unpredictability in working time and limiting their potential to improve workers’ temporal autonomy [40]. To advance employee well-being and retention, Korea may need to complement existing working-time regulations with stronger provisions for employee-centered schedule control, similar to the statutory right to request in the United Kingdom. Moreover, because many categories of workers remain outside the scope of current working-time protections, expanding coverage and ensuring robust enforcement and monitoring will be essential to make such rights meaningful in practice.

This study has several limitations. First, its cross-sectional design precludes definitive conclusions about causality. Although the KWSHS is a longitudinal panel survey, the multidimensional WTC scale was available only in the September 2024 wave, which limited our analysis to a cross-sectional framework. Consequently, we could not establish temporal precedence among WTC, burnout, and turnover intention. Longitudinal or experimental studies are needed to clarify directionality. Second, our measures were collected via a self-report survey and may have been subject to biases inherent to this method, such as common method bias and response tendencies. Third, although the sample was designed to reflect the demographic profile of Korean workers, it disproportionately included highly educated white-collar workers, who are generally more likely to participate in online panel surveys. As a result, the generalizability of the findings to low-wage service and blue-collar workers—who typically experience more rigid schedules and lower levels of WTC—may be limited. Importantly, WTC may be even more critical for these occupational groups, and their relative underrepresentation may have produced conservative estimates of the observed associations. In addition, several socio-demographic and job-related characteristics differed between included and excluded participants, suggesting potential selection bias due to missing data. However, the main analyses were adjusted for these characteristics, which may have partially mitigated this bias. Finally, our models did not incorporate other potentially relevant organizational factors, such as industry type or job rank, which could moderate or mediate the observed relationships.

Notably, although the prevalence of turnover intention was high (34.5%), the prevalence of burnout was relatively low (3.9%). This discrepancy is largely attributable to our strict operationalization of burnout, which required meeting clinical cutoffs across all 3 KBOSS dimensions. This conservative definition was intended to distinguish burnout as a distinct occupational syndrome in accordance with ICD-11 criteria, but it may have overlooked a substantial proportion of workers experiencing “moderate” or “early-stage” burnout. Our sensitivity analyses using alternative burnout definitions, including less stringent criteria and continuous burnout scores, yielded results consistent with the main findings, supporting the robustness of the observed associations while also underscoring the multidimensional nature of turnover intention.

Furthermore, while burnout was a significant mediator, it explained 36.6% of the association between WTC and turnover intention. This indicates that a substantial proportion of the effect (approximately 63.4%) may operate through other mechanisms. For example, low WTC might increase turnover intention by directly interfering with personal life and family responsibilities, independent of psychological exhaustion [36,41]. Other organizational factors, such as limited perceived organizational support or job dissatisfaction arising from rigid scheduling, may also play an important role [13,14]. Future research should examine these additional pathways to better capture the multifaceted impact of work-time autonomy on employee retention.

In this large cross-sectional study of Korean workers, low level of WTC was associated with higher burnout and greater turnover intention. Burnout mediated more than one-third of the association between WTC and turnover intention. These findings highlight the importance of strengthening employee-centered schedule control at both organizational and policy levels to protect worker well-being and reduce turnover.

## Figures and Tables

**Figure f1-epih-48-e2026011:**
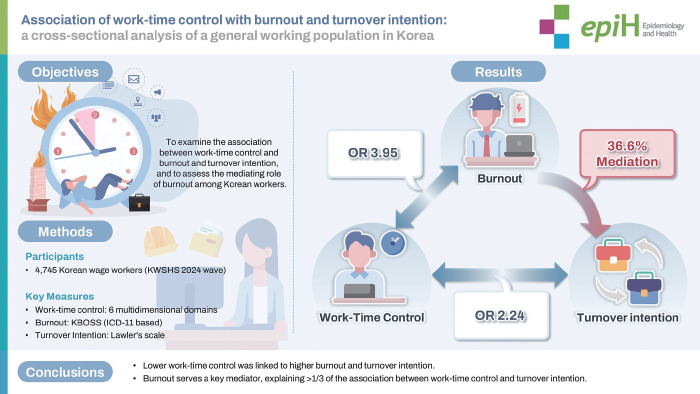


**Table 1.** Participant characteristics and prevalence of burnout and turnover intention

Characteristics	n (%)	Burnout		Turnover intention	
		n (row %)	p-value	n (row %)	p-value
Total	4,745 (100)	187 (3.9)		1,638 (34.5)	
Work-time control			<0.01		<0.01
Q1 (high)	1,016 (21.4)	22 (2.2)		278 (27.4)	
Q2	1,338 (28.2)	38 (2.8)		398 (29.8)	
Q3	1,274 (26.8)	46 (3.6)		460 (36.1)	
Q4 (low)	1,117 (23.5)	81 (7.3)		502 (44.9)	
Gender			0.05		<0.01
Men	2,483 (52.3)	85 (3.4)		775 (31.2)	
Women	2,262 (47.7)	102 (4.5)		863 (38.2)	
Age (yr)			<0.01		<0.01
20–29	887 (18.7)	54 (6.1)		380 (42.8)	
30–39	939 (19.8)	56 (6.0)		401 (42.7)	
40–49	1,154 (24.3)	45 (3.9)		411 (35.6)	
50–59	1,136 (23.9)	24 (2.1)		318 (28.0)	
≥60	629 (13.3)	8 (1.3)		128 (20.4)	
Education			0.56		<0.01
≤High school	618 (13.0)	27 (4.4)		204 (33.0)	
≥College	4,127 (87.0)	160 (3.9)		1,434 (34.8)	
Monthly salary (10⁴ KRW)			<0.01		<0.01
<200	320 (6.7)	9 (2.8)		104 (32.5)	
200–299	1,854 (39.1)	99 (5.3)		756 (40.8)	
300–399	1,340 (28.2)	51 (3.8)		467 (34.9)	
≥400	1,231 (25.9)	28 (2.3)		311 (25.3)	
Job			<0.01		0.89
White collar	3,566 (75.2)	135 (3.8)		1,233 (34.6)	
Pink collar	406 (8.6)	28 (6.9)		143 (35.2)	
Blue collar	773 (16.3)	24 (3.1)		262 (33.9)	
Working hours (hr/wk)			0.20		0.05
<40	772 (16.3)	22 (2.9)		241 (31.2)	
40–52	3,748 (79.0)	154 (4.1)		1,309 (34.9)	
>52	225 (4.7)	11 (4.9)		88 (39.1)	
Shift work			0.76		0.20
No	4,256 (89.7)	169 (4.0)		1,482 (34.8)	
Yes	489 (10.3)	18 (3.7)		156 (31.9)	

Q, quartile; KRW, Korean won.

**Table 2.** Associations of work-time control with burnout and turnover intention^1^

Work-time control	Unadjusted	Model 1	Model 2
Burnout			
Q1 (high)	1.00 (reference)	1.00 (reference)	1.00 (reference)
Q2	1.32 (0.78, 2.25)	1.39 (0.82, 2.38)	1.37 (0.80, 2.34)
Q3	1.69 (1.01, 2.83)	1.84 (1.10, 3.10)	1.80 (1.07, 3.03)
Q4 (low)	3.53 (2.19, 5.71)	4.03 (2.48, 6.56)	3.95 (2.41, 6.47)
Turnover intention			
Q1 (high)	1.00 (reference)	1.00 (reference)	1.00 (reference)
Q2	1.12 (0.94, 1.35)	1.17 (0.97, 1.40)	1.15 (0.95, 1.38)
Q3	1.50 (1.25, 1.79)	1.59 (1.33, 1.91)	1.53 (1.27, 1.84)
Q4 (low)	2.17 (1.81, 2.60)	2.38 (1.97, 2.87)	2.24 (1.85, 2.71)

Values are presented as odds ratio (95% confidence interval).

1 Model 1: Adjusted for gender and age; Model 2: Adjusted for gender, age, education, monthly salary, occupation, weekly working hours, and shift work.

**Table 3.** Mediating role of burnout in the association between work-time control and turnover intention (mediation analysis)

Variables	OR (95% CI)^1^
Total effect	1.96 (1.64, 2.28)
Direct effect	1.61 (1.40, 1.82)
Indirect effect	1.22 (1.09, 1.34)
Percentage mediated (%)	36.6 (22.3, 51.0)

OR, odds ratio; CI, confidence interval.

1 Adjusted for gender, age, education, monthly salary, occupation, weekly working hours, and shift work.

## Data Availability

The KWSHS data are available upon reasonable request, provided that researchers meet specific criteria.

## Supplementary materials

Supplementary Material 1. Odds ratio (95% confidence interval) of burnout by work-time control according to gender, age, and working hour

Supplementary Material 2. Odds ratio (95% confidence interval) of turnover intention by work- time control according to gender, age, and working hour

Supplementary Material 3. Odds ratios (95% confidence intervals) for burnout according to work-time control: sensitivity analysis using an alternative burnout definition^1^

Supplementary Material 4. Association between worktime control and continuous burnout score1 (KBOSS): sensitivity analysis

Supplementary Material 5. Associations of individual work-time control domains with burnout and turnover intention

Supplementary Material 6. Mediating role of burnout between WTC as continuous variable1 and turnover intention by mediation analysis

Supplementary Material 7. Baseline characteristics of included and excluded participants due to missing data
