# Supplementary material for: Association of work-time control with burnout and turnover intention: a cross-sectional analysis of a general working population in Korea
Source: Epidemiol Health. 2026 Feb 21;48:e2026011. doi: 10.4178/epih.e2026011 (PMC13033437; doi:10.4178/epih.e2026011)
Supplement: Supplementary Material 1. — Odds ratio (95% confidence interval) of burnout by work-time control according to gender, age, and working hour [file epih-48-e2026011-Supplementary-1.docx]

Supplementary Material 1. Odds ratio (95% confidence interval) of burnout by work-time control according to gender, age, and working hour

|  |  | Unadjusted | | Model 1^1^ | | Model 2^2^ | |
| --- | --- | --- | --- | --- | --- | --- | --- |
| **Gender** | **Men** | |  | |  | |  |
|  | Work-time control |  | |  | |  | |
|  | Q1 (high) | ref | | ref | | ref | |
|  | Q2 | 1.34 (0.66-2.75) | | 1.39 (0.78-2.85) | | 1.32 (0.64-2.73) | |
|  | Q3 | 1.44 (0.68-3.05) | | 1.57 (0.74-3.33) | | 1.56 (0.73-3.33) | |
|  | Q4 (low) | 4.02 (2.06-7.83) | | 4.78 (2.44-9.39) | | 4.65 (2.33-9.27) | |
|  | **Women** | |  | |  | |  |
|  | Work-time control |  | |  | |  | |
|  | Q1 (high) | ref | | ref | | ref | |
|  | Q2 | 1.29 (0.59-2.85) | | 1.40 (0.63-3.11) | | 1.41 (0.64-3.14) | |
|  | Q3 | 1.79 (0.86-3.72) | | 2.03 (0.97-4.23) | | 1.97 (0.94-4.12) | |
|  | Q4 (low) | 3.03 (1.51-6.07) | | 3.54 (1.76-7.14) | | 3.53 (1.74-7.17) | |
| **Age** | **20-29** | |  | |  | |  |
|  | Work-time control |  | |  | |  | |
|  | Q1 (high) | ref | | ref | | ref | |
|  | Q2 | 1.23 (0.50-3.02) | | 1.22 (0.50-3.00) | | 1.04 (0.41-2.60) | |
|  | Q3 | 1.80 (0.77-4.20) | | 1.83 (0.78-4.27) | | 1.67 (0.70-3.97) | |
|  | Q4 (low) | 3.09 (1.36-7.00) | | 3.18 (1.39-7.25) | | 2.97 (1.27-6.95) | |
|  | **30-39** | |  | |  | |  |
|  | Work-time control |  | |  | |  | |
|  | Q1 (high) | ref | | ref | | ref | |
|  | Q2 | 1.26 (0.49-3.27) | | 1.28 (0.50-3.33) | | 1.17 (0.44-3.06) | |
|  | Q3 | 1.75 (0.69-4.43) | | 1.70 (0.67-4.33) | | 1.60 (0.63-4.11) | |
|  | Q4 (low) | 4.06 (1.70-9.69) | | 4.16 (1.72-10.03) | | 4.12 (1.69-10.07) | |
|  | **40-49** | |  | |  | |  |
|  | Work-time control |  | |  | |  | |
|  | Q1 (high) | ref | | ref | | ref | |
|  | Q2 | 2.79 (0.78-9.98) | | 2.75 (0.77-9.85) | | 2.92 (0.81-10.55) | |
|  | Q3 | 2.82 (0.77-10.35) | | 2.62 (0.71-9.69) | | 2.60 (0.70-9.65) | |
|  | Q4 (low) | 5.95 (1.75-20.29) | | 5.41 (1.57-18.61) | | 5.73 (1.64-20.03) | |
|  | ≥**50** | |  | |  | |  |
|  | Work-time control |  | |  | |  | |
|  | Q1 (high) | ref | | ref | | ref | |
|  | Q2 | 0.71 (0.14-3.54) | | 0.70 (0.14-3.50) | | 0.75 (0.15-3.77) | |
|  | Q3 | 1.50 (0.39-5.85) | | 1.51 (0.39-5.90) | | 1.62 (0.41-6.41) | |
|  | Q4 (low) | 4.56 (1.34-15.54) | | 4.64 (1.35-15.92) | | 4.64 (1.32-16.37) | |
| **Working hours** | **<40** | |  | |  | |  |
| (h/wk) | Work-time control |  | |  | |  | |
|  | Q1 (high) | ref | | ref | | ref | |
|  | Q2 | 0.75 (0.19-3.04) | | 0.75 (0.18-3.05) | | 0.83 (0.20-3.42) | |
|  | Q3 | 0.87 (0.22-3.55) | | 0.99 (0.24-4.09) | | 1.06 (0.25-4.47) | |
|  | Q4 (low) | 2.34 (0.72-7.62) | | 2.66 (0.44-2.84) | | 3.31 (0.96-11.36) | |
|  | **40-52** | |  | |  | |  |
|  | Work-time control |  | |  | |  | |
|  | Q1 (high) | ref | | ref | | ref | |
|  | Q2 | 1.48 (0.83-2.65) | | 1.61 (0.90-2.87) | | 1.54 (0.86-2.76) | |
|  | Q3 | 1.74 (0.99-3.07) | | 1.90 (1.07-3.36) | | 1.82 (1.02-3.22) | |
|  | Q4 (low) | 3.44 (2.02-5.87) | | 3.93 (2.29-6.76) | | 3.69 (2.13-6.38) | |
|  | **>52^3^** | |  | |  | |  |
|  | Work-time control |  | |  | |  | |
|  | Q1 (high) | ref | | ref | | ref | |
|  | Q2 | - | | - | |  | |
|  | Q3 | - | | - | |  | |
|  | Q4 (low) | - | | - | |  | |

^1^Model 1 was adjusted for gender and age

^2^Model 2: Adjusted for gender, age, education, monthly salary, job, working hours, and shift work.

^3^Estimates for the ≥52 h/week group were not available due to sparse data.
