# Supplementary material for: Association of work-time control with burnout and turnover intention: a cross-sectional analysis of a general working population in Korea
Source: Epidemiol Health. 2026 Feb 21;48:e2026011. doi: 10.4178/epih.e2026011 (PMC13033437; doi:10.4178/epih.e2026011)
Supplement: Supplementary Material 2. — Odds ratio (95% confidence interval) of turnover intention by work- time control according to gender, age, and working hour [file epih-48-e2026011-Supplementary-2.docx]

Supplementary Material 2. Odds ratio (95% confidence interval) of turnover intention by work- time control according to gender, age, and working hour

|  |  | Unadjusted | | Model 1^1^ | | Model 2^2^ | |
| --- | --- | --- | --- | --- | --- | --- | --- |
| **Gender** | **Men** | |  | |  | |  |
|  | Work-time control |  | |  | |  | |
|  | Q1 (high) | ref | | ref | | ref | |
|  | Q2 | 1.16 (0.92-1.48) | | 1.18 (0.93-1.51) | | 1.17 (0.91-1.49) | |
|  | Q3 | 1.62 (1.26-2.07) | | 1.70 (1.32-2.18) | | 1.64 (1.28-2.13) | |
|  | Q4 (low) | 2.03 (1.57-2.64) | | 2.22 (1.70-2.89) | | 2.08 (1.59-2.72) | |
|  | **Women** | |  | |  | |  |
|  | Work-time control |  | |  | |  | |
|  | Q1 (high) | ref | | ref | | ref | |
|  | Q2 | 1.07 (0.81-1.41) | | 1.16 (0.88-1.55) | | 1.14 (0.86-1.52) | |
|  | Q3 | 1.31 (1.01-1.71) | | 1.52 (1.16-1.99) | | 1.43 (1.08-1.88) | |
|  | Q4 (low) | 2.08 (1.60-2.70) | | 2.53 (1.92-3.32) | | 2.34 (1.78-3.09) | |
| **Age** | **20-29** | |  | |  | |  |
|  | Work-time control |  | |  | |  | |
|  | Q1 (high) | ref | | ref | | ref | |
|  | Q2 | 1.09 (0.75-1.59) | | 1.11 (0.76-1.63) | | 1.06 (0.72-1.55) | |
|  | Q3 | 1.99 (1.37-2.88) | | 1.92 (1.32-2.80) | | 1.75 (1.19-2.56) | |
|  | Q4 (low) | 2.90 (1.94-4.34) | | 2.73 (1.82-4.09) | | 2.56 (1.69-3.87) | |
|  | **30-39** | |  | |  | |  |
|  | Work-time control |  | |  | |  | |
|  | Q1 (high) | ref | | ref | | ref | |
|  | Q2 | 1.10 (0.76-1.59) | | 1.08 (0.75-1.57) | | 1.05 (0.72-1.53) | |
|  | Q3 | 1.76 (1.20-2.56) | | 1.66 (1.13-2.42) | | 1.57 (1.07-2.31) | |
|  | Q4 (low) | 2.28 (1.52-3.41) | | 2.09 (1.39-3.14) | | 2.03 (1.34-3.07) | |
|  | **40-49** | |  | |  | |  |
|  | Work-time control |  | |  | |  | |
|  | Q1 (high) | ref | | ref | | ref | |
|  | Q2 | 1.10 (0.77-1.57) | | 1.08 (0.76-1.56) | | 1.06 (0.74-1.53) | |
|  | Q3 | 1.28 (0.89-1.86) | | 1.22 (0.84-1.78) | | 1.14 (0.78-1.67) | |
|  | Q4 (low) | 2.24 (1.55-3.22) | | 2.10 (1.45-3.03) | | 1.92 (1.32-2.80) | |
|  | ≥**50** | |  | |  | |  |
|  | Work-time control |  | |  | |  | |
|  | Q1 (high) | ref | | ref | | ref | |
|  | Q2 | 1.32 (0.92-1.89) | | 1.31 (0.91-1.88) | | 1.28 (0.89-1.85) | |
|  | Q3 | 1.60 (1.13-2.27) | | 1.67 (1.17-2.37) | | 1.59 (1.12-2.28) | |
|  | Q4 (low) | 2.45 (1.74-3.46) | | 2.61 (1.84-3.70) | | 2.31 (1.12-3.31) | |
| **Working hours** | **<40** | |  | |  | |  |
| (h/wk) | Work-time control |  | |  | |  | |
|  | Q1 (high) | ref | | ref | | ref | |
|  | Q2 | 1.13 (0.72-1.79) | | 1.11 (0.69-1.77) | | 1.13 (0.70-1.81) | |
|  | Q3 | 1.54 (0.97-2.43) | | 1.71 (1.06-2.77) | | 1.71 (1.05-2.79) | |
|  | Q4 (low) | 1.95 (1.24-3.08) | | 2.20 (1.36-3.54) | | 2.27 (1.39-3.69) | |
|  | **40-52** | |  | |  | |  |
|  | Work-time control |  | |  | |  | |
|  | Q1 (high) | ref | | ref | | ref | |
|  | Q2 | 1.13 (0.92-1.38) | | 1.19 (0.97-1.46) | | 1.15 (0.94-1.42) | |
|  | Q3 | 1.46 (1.20-1.78) | | 1.54 (1.25-1.88) | | 1.46 (1.19-1.79) | |
|  | Q4 (low) | 2.23 (1.82-2.74) | | 2.40 (1.95-2.96) | | 2.23 (1.80-2.76) | |
|  | **>52** | |  | |  | |  |
|  | Work-time control |  | |  | |  | |
|  | Q1 (high) | ref | | ref | | ref | |
|  | Q2 | 1.04 (0.44-2.79) | | 1.04 (0.43-2.51) | | 0.86 (0.34-2.17) | |
|  | Q3 | 2.05 (0.85-4.93) | | 2.10 (0.86-5.12) | | 1.99 (0.78-5.10) | |
|  | Q4 (low) | 1.87 (0.79-4.46) | | 2.13 (0.87-5.17) | | 2.03 (0.81-5.11) | |

^1^Model 1: Adjusted for gender and age

^2^Model 2: Adjusted for gender, age, education, monthly salary, job, weekly working hours, and shift work.
