# Supplementary material for: Association of work-time control with burnout and turnover intention: a cross-sectional analysis of a general working population in Korea
Source: Epidemiol Health. 2026 Feb 21;48:e2026011. doi: 10.4178/epih.e2026011 (PMC13033437; doi:10.4178/epih.e2026011)
Supplement: Supplementary Material 3. — Odds ratios (95% confidence intervals) for burnout according to work-time control: sensitivity analysis using an alternative burnout definition1 [file epih-48-e2026011-Supplementary-3.docx]

Supplementary Material 3. Odds ratios (95% confidence intervals) for burnout according to work-time control: sensitivity analysis using an alternative burnout definition^1^

| Work-time control | Unadjusted | Model 1^2^ | Model 2^3^ |
| --- | --- | --- | --- |
| Q1 (high) | ref | ref | ref |
| Q2 | 0.72 (0.58-0.90) | 0.75 (0.60-0.94) | 0.74 (0.59-0.93) |
| Q3 | 0.89 (0.72-1.10) | 0.93 (0.75-1.16) | 0.92 (0.74-1.15) |
| Q4 (low) | 1.30 (1.05-1.60) | 1.42 (1.14-1.76) | 1.39 (1.12-1.73) |

**^1^**Burnout was defined as meeting at least two of the three dimension-specific cut-offs of the Korean Burnout Syndrome Scale (KBOSS): exhaustion ≥21, cynicism ≥18, and reduced professional efficacy ≥15.

^2^Model 1: Adjusted for gender and age

^3^Model 2: Adjusted for gender, age, education, monthly salary, job, weekly working hours, and shift work.
