# Supplementary material for: Association of work-time control with burnout and turnover intention: a cross-sectional analysis of a general working population in Korea
Source: Epidemiol Health. 2026 Feb 21;48:e2026011. doi: 10.4178/epih.e2026011 (PMC13033437; doi:10.4178/epih.e2026011)
Supplement: Supplementary Material 4. — Association between worktime control and continuous burnout score1 (KBOSS): sensitivity analysis [file epih-48-e2026011-Supplementary-4.docx]

Supplementary Material 4. Association between worktime control and continuous burnout score^1^ (KBOSS): sensitivity analysis

| Work-time control | Mean (SD) | Unadjusted  β (95% CI) | Model 1^2^  β (95% CI) | Model 2^3^  β (95% CI) |
| --- | --- | --- | --- | --- |
| Q1 (high) | 41.8 (11.4) | ref | ref | ref |
| Q2 | 43.1 (10.6) | 0.2 (-0.9, 1.3) | 0.4 (-0.6, 1.5) | 0.4 (-0.7, 1.4) |
| Q3 | 42.9 (11.2) | 0.1 (-1.0, 1.2) | 0.5 (-0.6, 1.6) | 0.4 (-0.6, 1.5) |
| Q4 (low) | 45.0 (11.2) | 2.3 (1.2, 3.4) | 2.9 (1.8, 4.0) | 2.8 (1.7, 3.9) |

**^1^**Burnout score was treated as a continuous outcome using the total score of the Korean Burnout Syndrome Scale (KBOSS).

^2^Model 1: Adjusted for gender and age

^3^Model 2: Adjusted for gender, age, education, monthly salary, job, weekly working hours, and shift work.
