# Supplementary material for: Association of work-time control with burnout and turnover intention: a cross-sectional analysis of a general working population in Korea
Source: Epidemiol Health. 2026 Feb 21;48:e2026011. doi: 10.4178/epih.e2026011 (PMC13033437; doi:10.4178/epih.e2026011)
Supplement: Supplementary Material 5. — Associations of individual work-time control domains with burnout and turnover intention [file epih-48-e2026011-Supplementary-5.docx]

Supplementary Material 5. Associations of individual work-time control domains with burnout and turnover intention

| WTC domain^1^ | Burnout  OR (95% CI) | Turnover intention  OR (95% CI) |
| --- | --- | --- |
| Start/end times of the workday | 1.22 (1.08-1.37) | 1.16 (1.11-1.22) |
| Opportunities to take breaks | 1.35 (1.18-1.53) | 1.18 (1.04-1.37) |
| Handling private matters during work | 1.47 (1.27-1.70) | 1.20 (1.13-1.27) |
| Scheduling of shifts | 1.42 (1.23-1.64) | 1.23 (1.17-1.30) |
| Scheduling of vacations/paid days off | 1.37 (1.21-1.56) | 1.09 (1.03-1.15) |
| Taking unpaid leave | 1.21 (1.07-1.37) | 1.21 (1.15-1.28) |

^1^Each domain was analyzed separately as a continuous variable (per 1-point decrease), adjusted for gender, age, education, monthly salary, occupational class, weekly working hours, and shift work.
