# Supplementary material for: Association of work-time control with burnout and turnover intention: a cross-sectional analysis of a general working population in Korea
Source: Epidemiol Health. 2026 Feb 21;48:e2026011. doi: 10.4178/epih.e2026011 (PMC13033437; doi:10.4178/epih.e2026011)
Supplement: Supplementary Material 6. — Mediating role of burnout between WTC as continuous variable1 and turnover intention by mediation analysis [file epih-48-e2026011-Supplementary-6.docx]

Supplementary Material 6. Mediating role of burnout between WTC as continuous variable^1^ and turnover intention by mediation analysis

|  | OR (95% CI)^2^ |
| --- | --- |
| Total effect | 1.08 (1.06-1.10) |
| Direct effect | 1.05 (1.04-1.07) |
| Indirect effect | 1.02 (1.01-1.04) |
| Percentage mediated | 31.8% (20.0%-43.6%) |

^1^Work-time control was treated as a continuous variable using the total work-time control score with odds ratios estimated per one-point decrease in work-time control.

^2^Adjusted for gender, age, education, monthly salary, job, weekly working hours, and shift work.
