# Supplementary material for: Association of work-time control with burnout and turnover intention: a cross-sectional analysis of a general working population in Korea
Source: Epidemiol Health. 2026 Feb 21;48:e2026011. doi: 10.4178/epih.e2026011 (PMC13033437; doi:10.4178/epih.e2026011)
Supplement: Supplementary Material 7. — Baseline characteristics of included and excluded participants due to missing data [file epih-48-e2026011-Supplementary-7.docx]

Supplementary Material 7. Baseline characteristics of included and excluded participants due to missing data

|  | Included | Excluded due to missing data | p |
| --- | --- | --- | --- |
|  | (N=4745) | (N=592) |  |
| Gender |  |  |  |
| Men | 2483 (52.3) | 322 (54.4) | 0.34 |
| Women | 2262 (47.7) | 270 (45.6) |  |
| Age (years) |  |  |  |
| 20-29 | 887 (18.7) | 107 (18.1) | 0.12 |
| 30-39 | 939 (19.8) | 142 (24) |  |
| 40-49 | 1154 (24.3) | 131 (22.1) |  |
| 50-59 | 1136 (23.9) | 128 (21.6) |  |
| 60- | 629 (13.3) | 84 (14.2) |  |
| Education |  |  |  |
| ≤ High school | 618 (13.0) | 58 (9.8) | 0.03 |
| ≥ College | 4127 (87.0) | 534 (90.2) |  |
| Monthly salary (10,000KRW) |  |  |  |
| <200 | 320 (6.7) | 39 (6.6) | <0.01 |
| 200-299 | 1854 (39.1) | 178 (30.1) |  |
| 300-399 | 1340 (28.2) | 196 (33.1) |  |
| ≥400 | 1231 (25.9) | 179 (30.2) |  |
| Job |  |  |  |
| White collar | 3566 (75.2) | 517 (87.3) | <0.01 |
| Pink collar | 406 (8.6) | 23 (3.9) |  |
| Blue collar | 773 (16.3) | 52 (8.8) |  |
| Working hours (h/wk) |  |  |  |
| <40 | 772 (16.3) | 83 (14.0) | 0.17 |
| 40-52 | 3748 (79.0) | 487 (82.3) |  |
| >52 | 225 (4.7) | 22 (3.7) |  |
| Shift work |  |  |  |
| No | 4256 (89.7) | 571 (96.5) | <0.01 |
| Yes | 489 (10.3) | 21 (3.6) |  |

KRW, Korean won.
